# The NEDD8-activating enzyme inhibitor MLN4924 induces G2 arrest and apoptosis in T-cell acute lymphoblastic leukemia

**DOI:** 10.18632/oncotarget.8068

**Published:** 2016-03-14

**Authors:** Kun Han, Qingyang Wang, Huanling Cao, Guihua Qiu, Junxia Cao, Xin Li, Jing Wang, Beifen Shen, Jiyan Zhang

**Affiliations:** ^1^ Department of Molecular Immunology, Institute of Basic Medical Sciences, Beijing 100850, P. R. China

**Keywords:** neddylation, MLN4924, T-ALL, G2 arrest, apoptosis

## Abstract

The first-in-class compound MLN4924 is a small molecule inhibitor that selectively inactivates NEDD8-activating enzyme (NAE). The anticancer effects of MLN4924 have been attributed to impaired neddylation of Cullin proteins. Here, we show that treatment of T-cell acute lymphoblastic leukemia (T-ALL) cells with MLN4924 potently suppressed the neddylation of Cullins and the oncogenic growth of T-ALL cells *in-vitro*. Moreover, MLN4924 induced disease regression in an *in vivo* xenograft model. MLN4924 also induced cell cycle arrest at G2 phase and apoptosis in T-ALL cells. However, inhibition of the neddylation of Cullins alone could not explain the effects of MLN4924 in T-ALL cells. Gene expression profiling indicated ribosome function, steroid biosynthesis, and hematopoietic cell lineage pathways were affected by MLN4924 treatment. MLN4924 also induced nucleolar disruption, suggesting nucleolar stress signaling might contribute to the anticancer effects of MLN4924 in T-ALL cells. In addition, MLN4924 treatment reduced 14-3-3ξ\δ protein levels in T-ALL cells. Thus, MLN4924 may inhibit T-ALL cell proliferation via several pathways.

## INTRODUCTION

T-cell acute lymphoblastic leukemia (T-ALL) arises due to multiple genetic mutations in immature T cells that block differentiation, enhance survival, and lead to proliferation of malignant clones [1, 2]. Although intensified chemotherapy and bone marrow transplantation have improved survival rates, the 20% of T-ALL patients who relapse have little to no chance of a cure [1, 2]. A better understanding of oncogenic signaling pathways in T-ALL might improve treatments for such patients.

NEDD8 (neural precursor cell expressed developmentally downregulated protein 8) is the ubiquitin-like protein most homologous to ubiquitin [3, 4]. The covalent binding of NEDD8 to substrate proteins is called “neddylation”, and includes the following steps: mature NEDD8 is activated by NEDD8-activating enzyme E1 (NAE), transferred by NEDD8-conjugating enzyme E2, and conjugated to the substrate protein by a NEDD8-E3 ligase [3, 4]. The most well-characterized substrates of neddylation are the Cullin proteins, which are essential components of Skp1/Cullin/F-box protein (SCF)-like ubiquitin ligase complexes and play a pivotal role in ubiquitin-mediated proteolysis [5, 6]. SCF activity, which controls cell cycle progression, requires the neddylation of Cullins. Furthermore, SCF mediates the ubiquitination and subsequent degradation of inhibitor of κB (IκB) proteins, which sequester nuclear factor-κB (NF-κB) in the cytoplasm [5, 6]. Thus, neddylation is essential for NF-κB activity. Neddylation dysfunctions have been implicated in neurodegenerative diseases and cancer [3, 4].

The first-in-class compound MLN4924 is a small molecule inhibitor that selectively inactivates NAE. In tumor cells of epithelial origin, MLN4924 treatment results in uncontrolled S-phase DNA replication, leading to DNA damage and subsequent cell death through apoptosis [7–9]. Preclinical studies demonstrate that MLN4924 also plays a role in various hematologic malignancies, including myeloma, B-cell lymphoma, and acute myeloid leukemia [10–12]. Additionally, the accumulation of phosphorylated IκBα (P-IκBα), a known target of the NEDD8 pathway, interferes with NF-κB activity and is a key mechanism underlying the cell death-inducing effects of MLN4924 in hematologic malignancies [10–12]. Constitutive NF-κB activity also contributes to oncogenic growth in T-ALL cells [13–15]. However, the possible therapeutic role of MLN4924 in T-ALL has not been explored. In this work, we show that treatment with MLN4924 potently suppresses the neddylation of Cullins and oncogenic growth in T-ALL cells via additional, Cullin-independent mechanisms of action.

## RESULTS

### MLN4924 dose-dependently reduces the neddylation of Cullins in T-ALL cells

**Figure 1:**
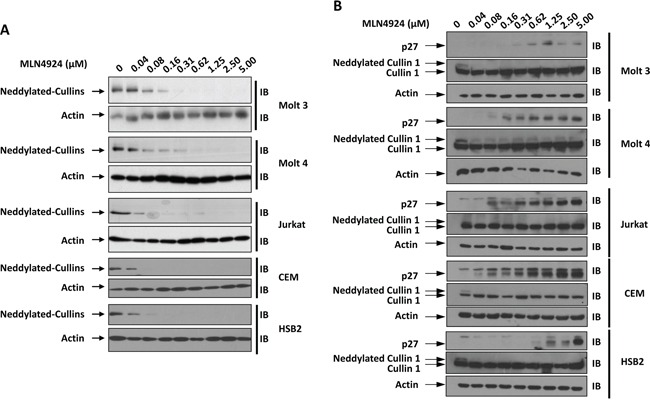
MLN4924 dose-dependently reduces the neddylation of Cullins in T-ALL cells T-ALL cell lines Molt 3, Molt 4, Jurkat, CEM, and HSB2 were treated with various doses of MLN4924 for 1 hour. Cell lysates were then prepared and used for immunoblotting (IB) with antibodies against NEDD8, p27, Cullin 1, and β-actin. Representative immunoblots from three independent experiments are shown.

Immunoblotting analysis of T-ALL cells with an antibody against NEDD8 revealed a major band at approximately 90-100 kDa (Figure 1A). Based on the molecular mass, the major band detected by the anti-NEDD8 antibody was composed of neddylated Cullins [12, 17–21]. MLN4924 dose-dependently suppressed the intensity of this band in all 5 T-ALL cell lines examined (Molt 3, Molt 4, Jurkat, CEM, and HSB2) as early as 1 hour after administration (Figure 1A). Immunoblotting with an antibody against Cullin 1 revealed the disappearance or dramatic decrease of the neddylated Cullin 1 band intensity after MLN4924 treatment (Figure 1B). p27, another substrate of SCF-like ubiquitin ligase [12], also accumulated in a dose-dependent manner after MLN4924 treatment (Figure 1B). Taken together, these observations suggest that MLN4924 potently inhibited the neddylation of Cullins.

### MLN4924 dose-dependently reduces oncogenic growth of T-ALL cells *in-vitro*

**Figure 2:**
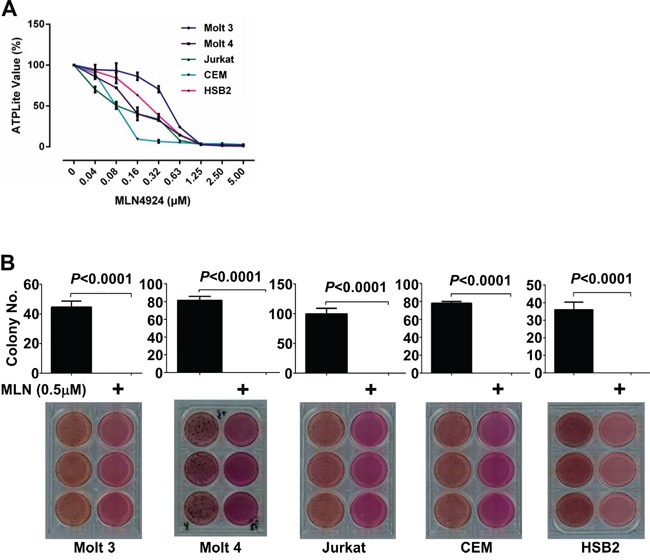
MLN4924 dose-dependently reduces the oncogenic growth of T-ALL cells *in-vitro* **A.** T-ALL cell lines Molt 3, Molt 4, Jurkat, CEM, and HSB2 were treated with various doses of MLN4924 for 72 hours. Cells were then subjected to ATPlite assays. **B.** T-ALL cells were cultured for 4 weeks in soft agar with 0.5 μmol/L MLN4924 or DMSO of equal volume. MTT was incorporated to make the colonies visible. Colonies were then counted. Data represent at least three independent experiments performed in triplicate.

Neddylation contributes to the oncogenic growth of various hematologic malignancies, including myeloma, B-cell lymphoma, and acute myeloid leukemia [10–12]. To investigate whether neddylation plays a similar role in T-ALL cells, Molt 3, Molt 4, Jurkat, CEM, and HSB2 cells were treated with varying concentrations of MLN4924. The growth of T-ALL cells was examined with ATPlite assays. As expected, MLN4924 dose-dependently inhibited T-ALL cell growth (Figure 2A). At doses higher than 1.25 μmol/L, >95% inhibition was observed in all T-ALL cell lines (Figure 2A). We chose the 0.5 μmol/L dose of MLN4924 for subsequent experiments.

We then tested the effect of MLN4924 treatment on the colony forming ability of T-ALL cells. Soft-agar assays showed that all T-ALL cell lines failed to form colonies in the presence of 0.5 μmol/L MLN4924, even though they readily formed colonies in the absence of MLN4924 (Figure 2B).

### MLN4924 induces disease regression in an *in-vivo* xenograft model

**Figure 3:**
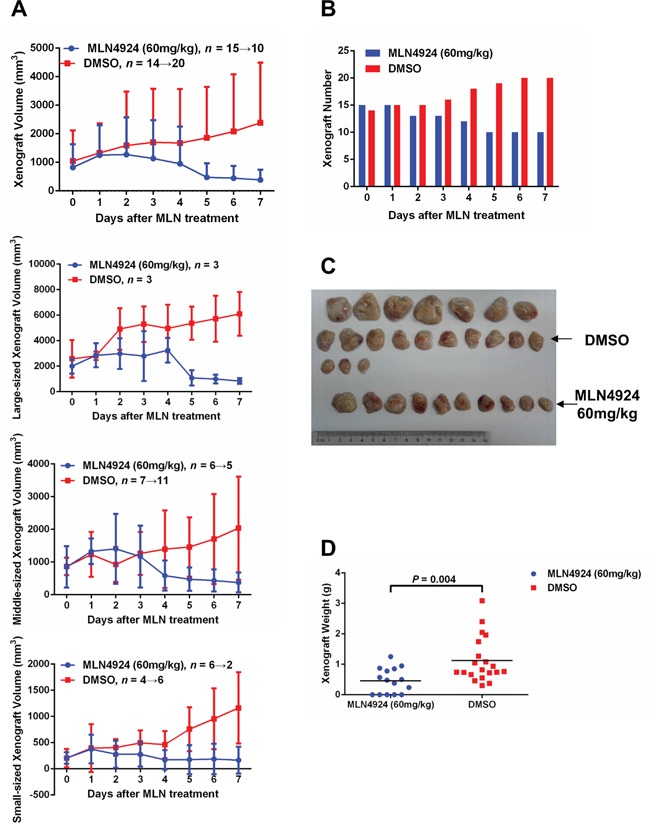
MLN4924 induces disease regression in an *in-vivo* xenograft model An *in-vivo* xenograft model was established with CEM cells. 6 weeks after inoculation, 12 NOD/SCID mice (each with 4 subcutaneous inoculations) were randomized into two groups with similar tumor numbers and volumes. MLN4924 (60 mg/kg) or DMSO of equal volume was administrated intraperitoneally once every day for 7 days. The growth curves **A.** numbers **B.** images **C.** and weights **D.** of subcutaneous tumors are shown. In the MLN4924 group, 5 tumors completely disappeared after 7 days of therapy, whereas in DMSO group, 6 new tumors appeared during the same period.

To determine whether MLN4924 has *in-vivo* anticancer activity in T-ALL cells, an *in-vivo* xenograft model was established with CEM cells. 12 NOD/SCID mice were used in total, and each mouse was given 4 subcutaneous inoculations. After 6 weeks, the mice were randomized into two groups with similar tumor numbers and volumes. One group (6 mice with 15 tumors on day 0 of treatment) was given MLN4924 at a dose of 60 mg/kg once a day for 7 days, and the other group (6 mice with 14 tumors on day 0) was given an equal volume of DMSO under the same schedule. MLN4924 treatment impaired tumor growth, as revealed by both tumor growth curves (Figure 3A, Supplementary Table 1) and xenograft weights (Figure 3C, 3D). Moreover, 5 tumors completely disappeared after 7 days of MLN4924 therapy (Figure 3B). By contrast, in the DMSO group, all xenografts continued growing and 6 new tumors appeared during this period (Figure 3). Thus, neddylation is essential for the oncogenic growth of T-ALL cells both *in-vitro* and *in-vivo*.

### MLN4924 induces cell cycle arrest at G2 phase in T-ALL cells

**Figure 4:**
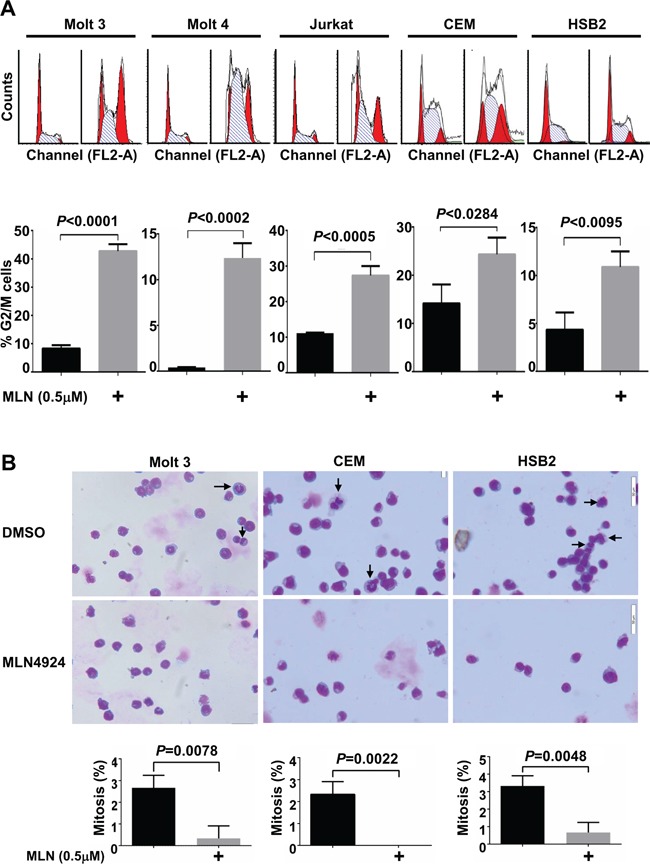
MLN4924 induces cell cycle arrest in the G2 phase in T-ALL cells **A.** and **B.** T-ALL cells were treated with 0.5 μmol/L MLN4924 or DMSO of equal volume for 24 hours. Cells were then subjected to cell cycle analysis by propidium iodide staining (A) or Giemsa staining (B). Arrows point to cells in mitosis. Data represent at least three independent experiments performed in triplicate.

Previous studies have revealed that MLN4924 treatment decreases the growth of various malignant cells by causing cell cycle arrest and/or apoptosis. Hence, we determined whether MLN4924 suppressed the growth of T-ALL cells via the same mechanism. Cell cycle analysis revealed that G2/M arrest increased in all 5 T-ALL cell lines after treatment with 0.5 μmol/L MLN4924 for 24 hours (Figure 4A). However, no DNA re-replication was observed. Despite this increase in the G2/M population, Giemsa staining revealed the absence of mitosis after MLN4924 treatment (Figure 4B). Thus, MLN4924 treatment of T-ALL cells leads to cell cycle arrest in the G2 phase.

### MLN4924 induces apoptosis in T-ALL cells

**Figure 5:**
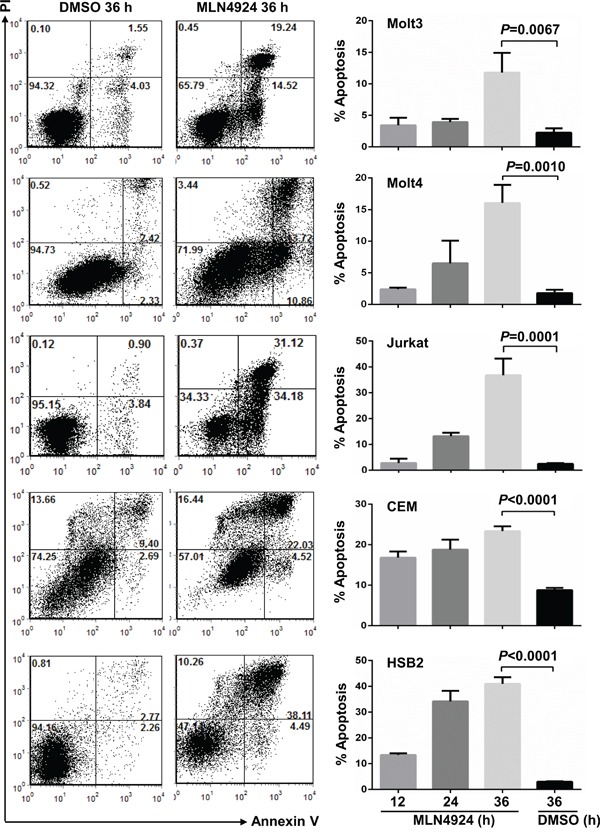
MLN4924 induces apoptosis in T-ALL cells T-ALL cells were treated with 0.5 μmol/L MLN4924 for 0, 12, 24, or 36 hours. Cells were then subjected to apoptosis analysis by AnnexinV staining. Data represent at least three independent experiments performed in triplicate.

Consistent with the absence of the sub-G1 cell population, an indicator apoptosis, in the cell cycle analysis, Annexin V/PI and Annexin V/7-AAD staining revealed that 0.5 μmol/L MLN4924 treatment marginally reduced survival in most T-ALL cell lines tested (except HSB2) for up to 24 hours (Figure 5). However, 0.5 μmol/L MLN4924 treatment for 36 hours significantly increased apoptosis (Figure 5). Therefore, MLN4924 eventually induces apoptosis in T-ALL cells.

### Cullins only partially mediate the effects of MLN4924 in T-ALL cells

**Figure 6:**
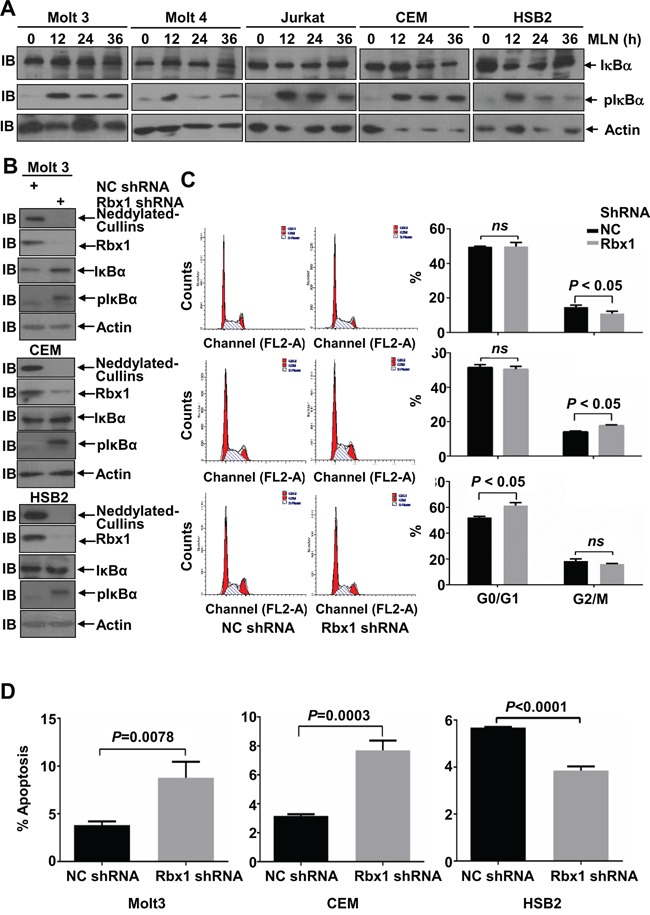
Cullins only partially mediate the effects of MLN4924 in T-ALL cells **A.** T-ALL cells were treated with 0.5 μmol/L MLN4924 for 0, 12, 24, or 36 hours. Cell lysates were then prepared and used for immunoblotting with antibodies against IκBα, P-IκBα, and β-actin. **B-D.** T-ALL cells were infected with lentivirus expressing non-targeting control shRNA or Rbx1 shRNA for 96 h. Cell lysates were then harvested and used for either immunoblotting with the indicated antibodies (**B**); cell cycle analysis by propidium iodide staining (**C**); or apoptosis analysis by AnnexinV staining (D). Data represent at least three independent experiments performed in triplicate.

Immunoblotting analysis revealed that MLN4924 treatment led to the accumulation of P-IκBα in all 5 T-ALL cell lines, although only weakly in Molt 4 cells (Figure 6A). However, no significant accumulation of IκBα, the substrate of SCF-like ubiquitin ligase [5, 6], was observed in T-ALL cells under the same conditions (Figure 6A). These data suggest that neddylation of Cullins only marginally hinders IκBα protein stability in T-ALL cells. Because the neddylation of most Cullins depends on RING-box protein 1 (Rbx1) [22], we infected Molt 3, CEM, and HSB2 cells with lentiviral constructs expressing Rbx1 short hairpin RNA (shRNA). As expected, silencing endogenous Rbx1 expression both reduced the neddylation of Cullins and caused P-IκBα accumulation (Figure 6B). Under the same conditions, weak accumulation of IκBα was observed in Molt 3 and CEM cells, but not in HSB2 cells (Figure 6B). These changes were not associated with an increase in the G2/M population in any of the 3 cell lines tested (Figure 6C). Thus, the neddylation system also promotes cell cycle progression independently of Cullins. On the other hand, Rbx1 knockdown was associated with increased apoptosis in Molt 3 and CEM cells, but not in HSB2 cells (Figure 6D). Therefore, the pro-apoptotic effect of MLN4924 is dependent, at least partially, on Cullins in Molt 3 and CEM cells, even though NF-κB is unlikely to be involved.

### Transcriptional profiles of MLN4924-treated T-ALL cells

**Figure 7:**
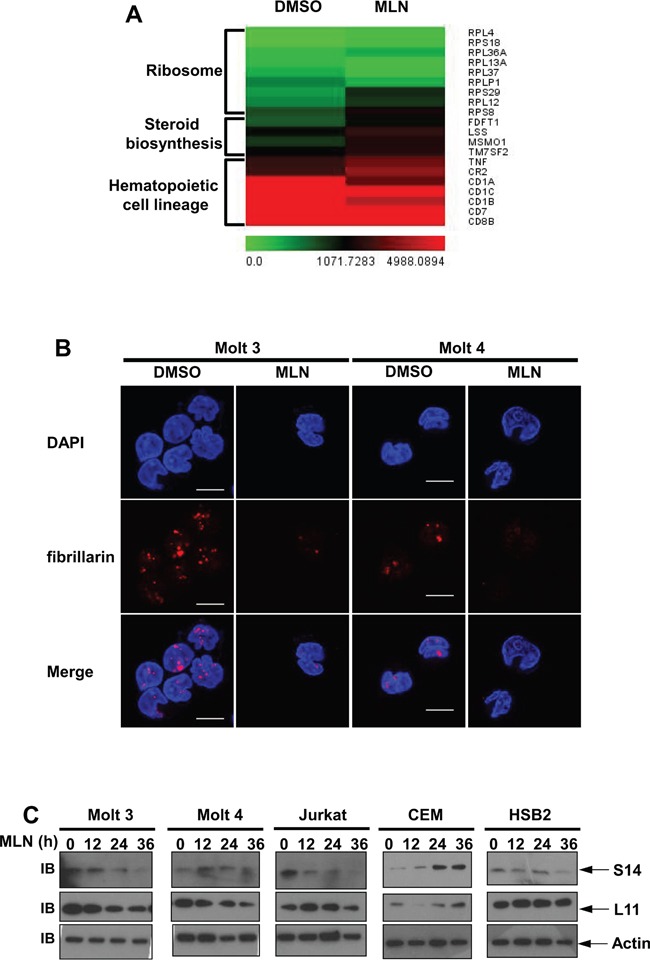
Transcriptional profiles of MLN4924-treated T-ALL cells **A.** Molt 3 cells were treated with 0.5 μmol/L MLN4924 or DMSO of equal volume for 24 hours. MLN4924-triggered transcriptional gene responses were analyzed by microarray. Affected genes involved in ribosome funciton, steroid biosynthesis, and hematopoietic cell lineages are shown in heatmaps. Mean values from three independent experiments are shown. **B.** T-ALL cells were treated with 0.5 μmol/L MLN4924 or DMSO of equal volume for 24 hours. Cells were then used for immunofluorescence analysis of fibrillarin expression, scale bar 10 μm. **C.** T-ALL cells were treated with 0.5 μmol/L MLN4924 for 0, 12, 24, or 36 hours. Cell lysates were then prepared and used for immunoblotting with antibodies against β-actin and ribosomal proteins S14 and L11.

Next, we used gene expression profiling to determine which pathways were deregulated in Molt 3 cells after MLN4924 treatment. 21,340 of the genes incorporated in the probe set were expressed in these cells. Using a cutoff of log_2_|fold change| ≥ 0.585, we identified 224 upregulated and 248 downregulated genes after MLN4924 treatment (*P* < 0.05). In line with our previous data, none of the 472 altered genes were NF-κB target genes. Of the 472 altered genes, pathway analysis revealed that 9 ribosome genes, 4 steroid biosynthesis genes, and 7 hematopoietic cell lineage genes were enriched (Figure 7A). Aberrant expression of ribosomal proteins frequently leads to nucleolar stress [23, 24]. Indeed, immunofluorescence analysis with an antibody against fibrillarin, a marker of nucleoli [25], revealed that 0.5 μmol/L MLN4924 treatment resulted in nucleolar disruption in all of the T-ALL cell lines examined (Figure 7B and Supplementary Figure 1). Transcriptional profiles of ribosomal proteins L11 and S14 [26, 27], which are neddylation substrates involved in nucleolar stress signaling, were not changed after MLN4929 treatment (Figure 7A). However, 0.5 μmol/L MLN4924 treatment decreased L11 and/or S14 protein levels in all T-ALL cell lines except CEM (Figure 7C). On the contrary, S14 accumulated in CEM cells after MLN4924 treatment (Figure 7C). Thus, it is possible that changes in the mRNA levels of the ribosome genes shown in Figure 7A can be attributed to changes in L11 and/or S14 protein levels. Together, these data suggest that nucleolar stress signaling might contribute to the effects of MLN4924 in T-ALL cells.

### MLN4924 treatment reduces 14-3-3ξ\δ protein levels in T-ALL cells

**Figure 8:**
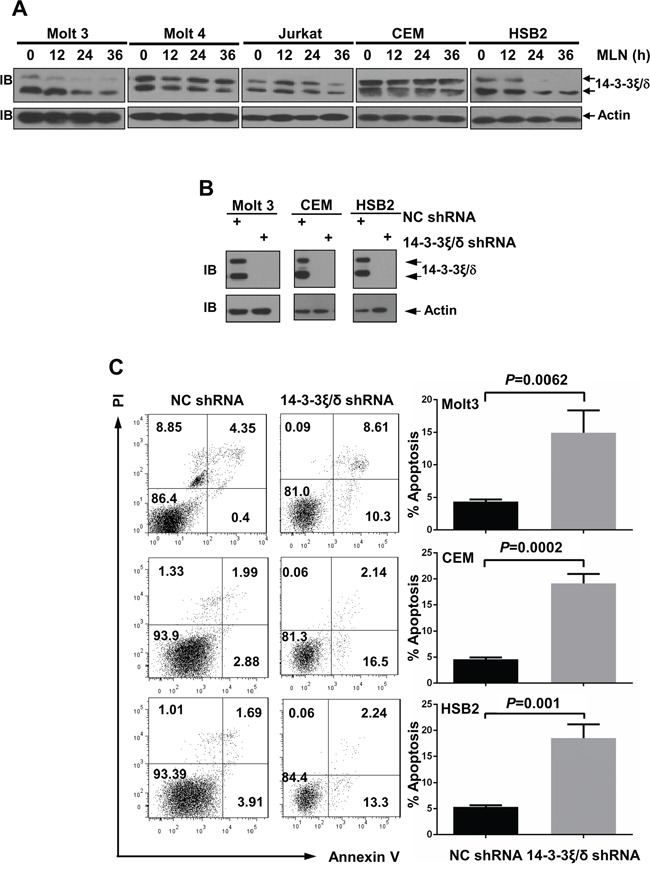
MLN4924 treatment reduces 14-3-3ξ\δ protein levels in T-ALL cells **A.** T-ALL cells were treated with 0.5 μmol/L MLN4924 for 0, 12, 24, or 36 hours. Cell lysates were then prepared and used for immunoblotting with antibodies against 14-3-3ξ\δ and β-actin. **B, C.** T-ALL cells were infected with lentivirus expressing non-targeting control shRNA or 14-3-3ξ\δ shRNA for 96 h. Cell lysates were then harvested and used for either immunoblotting with the indicated antibodies (B) or apoptosis analysis by AnnexinV staining (C). Data represent at least three independent experiments performed in triplicate.

We also tested several proteins involved in apoptosis. Immunoblotting analysis revealed that levels of 14-3-3ξ\δ proteins, important scaffold proteins that prevent bcl2 family member-induced apoptosis [28], decreased in T-ALL cells after MLN4924 treatment (Figure 8A). Larger decreases (Figure 8A) were associated with larger increases in apoptosis (Figure 5). Because efficient knockdown of 14-3-3ξ\δ (Figure 8B) increased apoptosis in Molt 3, CEM, and HSB2 cells (Figure 8C), MLN4924 might inhibit the survival of T-ALL cells at least in part by reducing 14-3-3ξ\δ protein levels.

## DISCUSSION

Our work suggests that MLN4924 might be effective in treating T-ALL. MLN4924 suppressed oncogenic growth in T-ALL cells by inducing cell cycle arrest at the G2 phase and late-onset apoptosis. Furthermore, analysis of transcriptional profiles suggests that MLN4924 treatment increases the expression of some genes involved in immune response (Supplementary Figure 2). The upregulation of genes encoding effector molecules such as *GZMA* and *TNF* suggests that T-ALL cells might gain certain effector functions after MLN4924 treatment. The partial differentiation of these T-ALL cells after MLN4924 treatment might contribute to a cure for this disease.

More importantly, our work indicates that nucleolar stress signaling might contribute to the effects of MLN4924 in T-ALL cells. Nucleolar stress signaling might induce cell cycle arrest and apoptosis through p53-dependent and p53-independent pathways [23]. Since p53 is frequently nonfunctional in T-ALL cells, and p53 target genes were not enriched among the 472 changed genes identified by microarray here, MLN4924-induced nucleolar stress signaling most likely increases cell cycle arrest and apoptosis through p53-independent pathways.

Our data also suggest novel roles of the neddylation system. Here, neddylation maintained levels of the scaffold protein 14-3-3ξ\δ. Since MLN4924 treatment did not decrease 14-3-3ξ\δ mRNA levels (Supplementary Figure 2), it is possible that the neddylation system prevents the degradation of 14-3-3ξ\δ through as yet unidentified mechanism(s). Furthermore, our microarray data suggest that the neddylation system is involved in steroid biosynthesis. These possible novel effects of the neddylation system in T-ALL and the underlying mechanisms require further study.

## MATERIALS AND METHODS

### Cell culture and transduction

The human T-ALL cell lines Molt 3, Molt 4, Jurkat, CEM and HSB2, were gifts from Dr. Ben Chen (Division of Hematology and Oncology, Department of Internal Medicine and Karmanos Cancer Institute, Wayne State University School of Medicine) and were grown in RPMI 1640 containing 10% fetal bovine serum, 2 mmol/L L-glutamine, 100 units/mL penicillin, and 100 μg/mL streptomycin. These cells were obtained and characterized in 1995 according to antigen/receptor expression. After that, the cells were frozen in multiple stocks until they were used in this work. For each cell line used, cell stock was made within 5 to 7 days of resuscitation. The continuous passage time was always kept within 1 to 2 months. Transduction was performed with lentivirus (multiplicity of infection=100). Lentivirus-based Rbx1 shRNA (GGGCAAGAAGCGCTTTGAAGT), 14-3-3ξ/δ shRNA (GCTGGTTCAGAAGGCCAAACT), and non-targeting control shRNA were from Shanghai GeneChem.

### Reagents

MLN4924 was purchased from Active Biochem. Antibody against NEDD8 was purchased from Cell Signaling Technology. Antibodies against Cullin 1, p27, IκBα, β-actin, and ribosomal proteins S14 and L11 were from Santa Cruz. Antibodies against Rbx1 and fibrillarin were from Abclonal. Antibody against 14-3-3ξ\δ was from BioLegend. The annexinV apoptosis detection kit was from BD Bioscience. The ATPlite 1 step kit was from Perkin Elmer. MTT, RNase A, and propidium iodide (PI) were from Sigma.

### Colony forming assays

T-ALL cells were seeded into 6-well plates at a density of 2×10^3^/well. The final concentration of MLN4924 was 0.5 μmol/L. DMSO was added to keep concentrations of DMSO (<0.1%) equal in all the wells. Cells were cultured for 4 weeks in soft-agar. Then, MTT was incorporated to make the colonies visible.

### *In-vivo* xenograft experiment

Male non-obese diabetic/severe combined immunodeficient (NOD/SCID) mice were purchased from Institutes of Experimental Animals, Academy of Chinese Medical Sciences and maintained under specific pathogen-free conditions. All experiments were performed in accordance with institutional guidelines for animal care. The xenograft experiment was carried out 12 in six-to-eight-week-old NOD/SCID mice with CEM cells according to the reported protocol [29]. Each mouse was given 4 inoculations along the back. 6 weeks after inoculation, tumor length (marked as L, mm) and width (marked as W, mm) were measured and volume (mm^3^) was calculated using the equation for an ellipsoid (L×W^2^×π/6). Meanwhile, xenografts were classified into 3 categories: large, with both L and W greater than 15mm; small, with both L and W less than 10mm; or middle for all other measurements.

The 12 mice were divided into 2 groups and each mouse was labeled with an ear mark so that every xenograft was traceable. Randomization was conducted to ensure that both groups had similar numbers of tumors in total (15:14) and in each category (Large, 3:3; middle, 6:7; and small, 6:4).

To determine whether MLN4924 had *in-vivo* anticancer activity in T-ALL cells, one group was intraperitoneally injected with MLN4924 at a dose of 60 mg/kg once every day for 7 days, and the other 6 mice were given DMSO injections of equal volume at the same times. The sizes of subcutaneous tumors were recorded every day during MLN4924 treatment.

### Cell cycle analysis

Cells were treated with 0.5 μmol/L MLN4924 or DMSO of equal volume for 24 hours. 1×10^6^ cells were harvested and fixed in 75% cold ethanol for at least 18 hours, digested with RNase A (10 μg/mL, 30 min) at 37¼C, labeled with PI (50 μg/mL, 30 min) at room temperature in the dark, and analyzed by flow cytometry. Flow cytometry was carried out on a Becton Dickinson FACS Calibur machine (BD Biosciences).

### Apoptosis analysis

Cells were treated with 0.5 μmol/L MLN4924 or DMSO of equal volume for 12, 24 and 36 hours. 1×10^5^ cells were then harvested and labeled with Annexin V, PI, or 7-aminoactinomycin D (7AAD), and analyzed by flow cytometry.

### ATPlite assays

Cells (1×10^4^ cells/well) were seeded in 96-well plates and treated with various doses of MLN4924 as indicated. DMSO was added to keep concentrations of DMSO (<0.1%) equal in all wells. After 72 hours, cell growth was assessed by ATPlite assays according to the manufacturer's protocol.

### Immunoblotting analysis

The cells were treated under various conditions as indicated in the figure legends and were subjected to immunoblotting analysis as described previously [16].

### Microarray analysis

RNA amplified using the OneArray Amino Allyl aRNA Amplification Kit (Phalanx Biotech Group) was labeled with Cy5 dyes (Amersham) and hybridized to Mouse Whole Genome OneArray with Phalanx hybridization buffer using the Phalanx Hybridization System. The data were analyzed according to the manufacturer's protocol.

### Immunofluorescence

Cells were treated with 0.5 μmol/L MLN4924 or DMSO of equal volume for 24 hours. Immunofluorescence analysis was then performed as described previously [30].

### Giemsa staining

Cells were collected on pre-coated (poly-L-lysine) coverslips, fixed with methanol, and stained with Giemsa dye for 10 min. After washing with water, cell morphology was observed under a microscope.

### Statistical analysis

The data are shown as mean ± standard deviations (SD). Student's *t*-test was employed to determine significant differences between two groups (paired or unpaired), and one-way ANOVA was used to determine significant differences among several groups. Differences were considered statistically significant when *P* < 0.05.

## SUPPLEMENTARY FIGURES AND TABLE




