# Supplementary material for: The NEDD8-activating enzyme inhibitor MLN4924 induces G2 arrest and apoptosis in T-cell acute lymphoblastic leukemia
Source: Oncotarget. 2016 Mar 14;7(17):23812–24. doi: 10.18632/oncotarget.8068 (PMC5029665; doi:10.18632/oncotarget.8068)
Supplement: Supplementary file 1 [file oncotarget-07-23812-s001.pdf]

SUPPLEMENTARY FIGURES AND TABLE

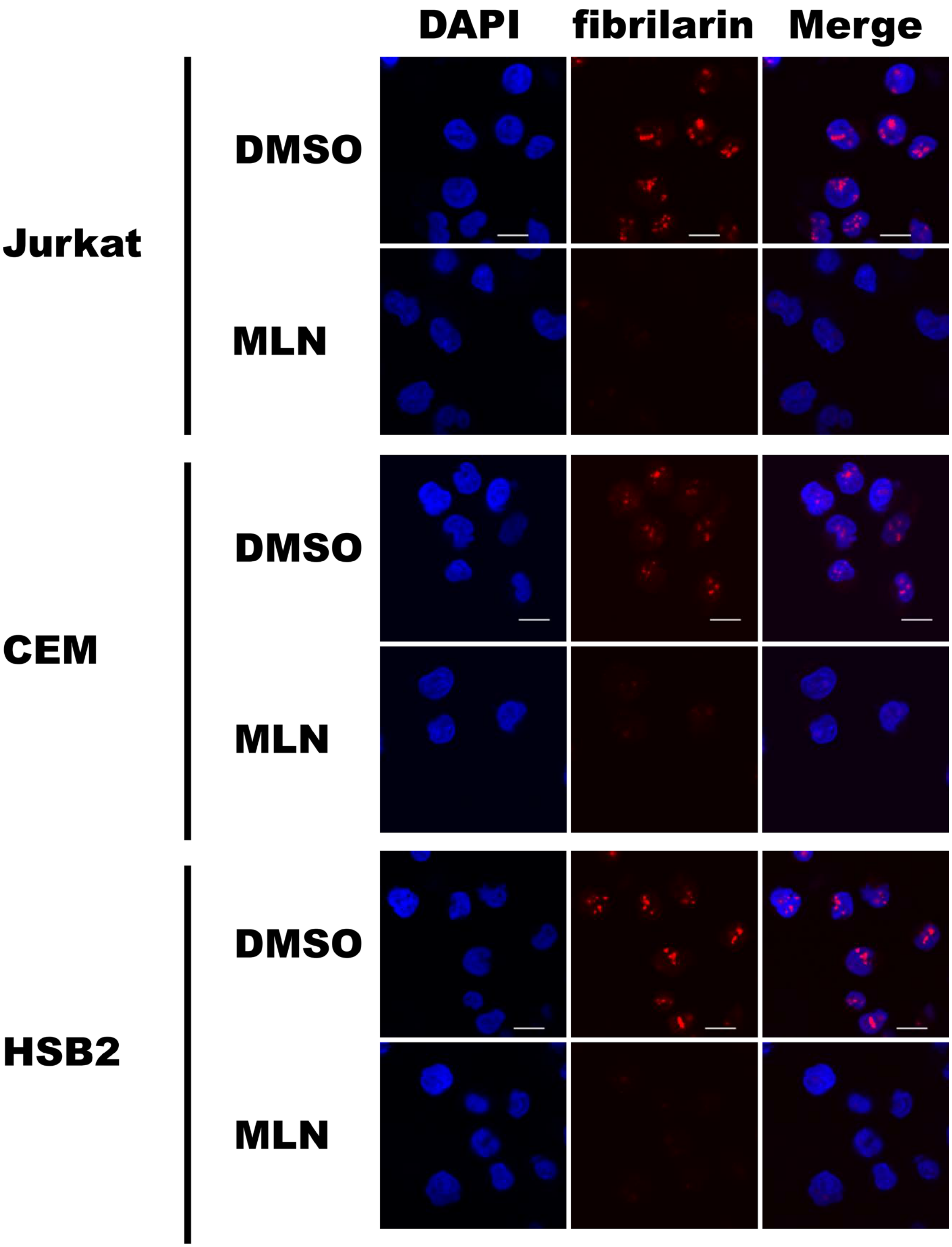

Supplementary Figure S1: Immunofluorescence analysis of fibrillarin expression of Jurkat, CEM and HSB2 cells. Cells were treated with 0.5 µmol/L MLN4924 or DMSO of equal volume for 24 hours, then used for immunofluorescence analysis of fibrillarin expression; scale bar 10 µm.

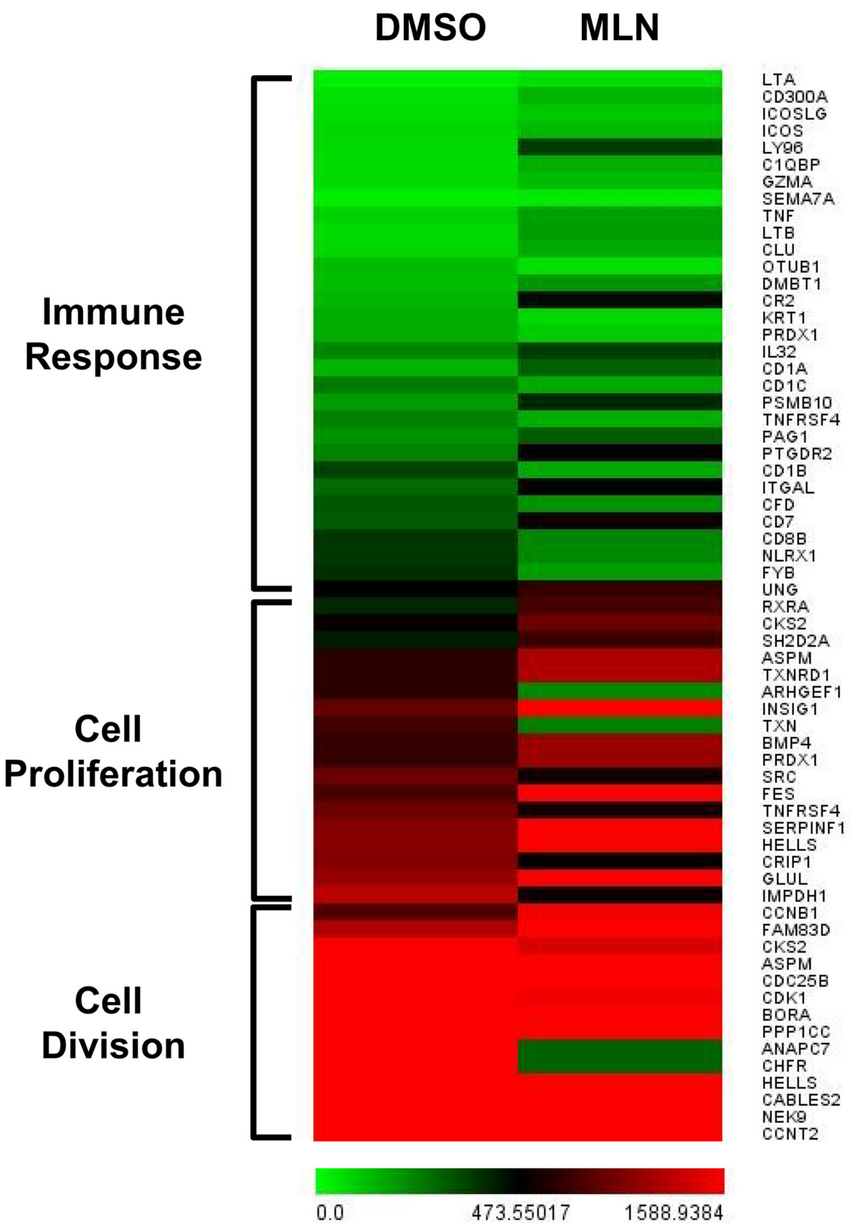

Supplementary Figure S2: Transcriptional profiles of MLN4924-treated T-ALL cells. Molt 3 cells were treated with 0.5 μmol/L MLN4924 or DMSO of equal volume for 24 hours. Affected genes involved in immune response, cell proliferation, and cell division are shown in heatmaps. Mean values of three independent experiments are shown..

Supplementary Table S1: Raw data from the *in-vivo* xenograft model. 12 NOD/SCID mice were identified with ear marks numbered 14476 to 14487. LEFT UP, LEFT DOWN, RIGHT UP, and RIGHT DOWN indicate the 4 inoculation sites along the back. The sizes of subcutaneous tumors were recorded every day before and during MLN4924 or DMSO treatment. “na” means that no xenograft was detectable, “/” means that xenografts disappeared, and bold figures in red represent newly-developed xenografts.

See Supplementary File S1
